# The influence of spray drying parameters and carrier material on the physico-chemical properties and quality of chokeberry juice powder

**DOI:** 10.1007/s13197-019-04088-8

**Published:** 2019-09-12

**Authors:** Magdalena Agnieszka Bednarska, Emilia Janiszewska-Turak

**Affiliations:** Department of Food Engineering and Process Management, Warsaw University of Life Sciences (SGGW), Nowoursynowska 159c, 02-776 Warsaw, Poland

**Keywords:** Anthocyanin, Arabic gum, Storage, Colour, Morphology

## Abstract

Chokeberry juice is used in the food industry because of its antioxidant activity as well as anti-diabetes, anti-mutagenic, bacteriostatic, anti-inflammatory and anti-virus properties. However in a liquid form its active ingredients can be unstable, so conversion into powder form is required. To improve the quality of the final product and extend information about the properties of chokeberry powder the aim of the study was to examine the relationship of inlet air temperature (160 °C, 200 °C) and carrier type maltodextrin (MD) 10DE and 15.6DE, arabic gum (AG) and their mixtures (AG:MD10 and AG:MD15—1:1, 1:3, 3:1) with the amounts of anthocyanins and polyphenols and selected physical properties of powders obtained after spray drying. Moreover stability of anthocyanin was tested after storage Obtained powders were characterized by low water activity (< 0.26), high dry matter content (97–99%) and good hygroscopic properties. Saccharification level of maltodextrin had no impact on the amount of bioactive components enclosed inside the capsules. Moreover, the mixtures of carriers, AG:MD appeared to have a large potential to ensure a high quality of chokeberry powder with high content of anthocyanin (1694–2028 mg/100 g) and polyphenols (about 3000 mg/100 g d.m.). Storage temperature mostly has no statistically significant influence on content of active ingredients, but an increase in colour coefficents was observed. The contents of anthocyanins and polyphenols were higher in powders stored at 4 °C than at 25 °C.

**Electronic supplementary material:**

The online version of this article (10.1007/s13197-019-04088-8) contains supplementary material, which is available to authorized users.

## Introduction

Chokeberry fruits are used in the food and pharmaceutical industry for their valuable properties, including antioxidant activity preventing diabetes, anti-mutagenic, bacteriostatic, anti-inflammatory and anti-virus properties (Samoticha et al. 2016). All those properties are a result of a large amount of anthocyanins, phenol acids and flavonoids and a relatively large amount of vitamin C (Błaszczak et al. 2017; Horszwald et al. 2013). The most common anthocyanins found in chokeberry are cyanidin-3-galactoside and cyanidin-3-arabinoside (Horszwald et al. 2013; Samoticha et al. 2016). However, despite their benefits, they are occasionally consumed as fresh, because of their specific sour and bitter taste, considered by consumers as negative. That is the reason for processing chokeberry into juices, syrups, jams, dyes, extracts, wines and teas (Horszwald et al. 2013; Błaszczak et al. 2017; Samoticha et al. 2016). However, thermal methods of processing products can decrease product quality (Błaszczak et al. 2017).

One method used for limitating loss of food components and protecting themfrom environmental factors is spray drying with additional materials providing protection and the possibility to obtain powder particles (carrier materials). Carrier material should meet some basic requirements such as: high molecular weight, high solubility, no reaction with the tested substance and low viscosity in the created solutions. Usually there is no single carrier material which can provide all those requirements, so to improve microencapsulation efficiency and protective capacity a mixture of them, in different percentages, is usually used (Jafari et al. 2017; Janiszewska-Turak et al. 2017; Santhalakshmy et al. 2015).

The most commonly used materials are maltodextrins with different dextrose equivalent, Arabic gum and modified starch. The use of these carriers or their mixtures in different proportions, can result in powders with different physical properties. Moreover, encapsulation efficiency can be influenced by those carriers (Mahdavi et al. 2016). Maltodextrin is used because of its higher water solubility, low viscosity and near transparency in solutions. Arabic gum is chosen because of creating stable emulsions, high solubility and pH stability (Janiszewska-Turak et al. 2017; Mahdavi et al. 2016). Chokeberry juice encapsulation by different methods with carriers, mostly only maltodextrin was performed and evaluated by some researchers (Gawałek et al. 2017; Pieczykolan and Kurek 2019; Wilkowska et al. 2017). The most commonly used proportion of carriers in its mixture, for encapsulation of fruit or vegetable juices, was 1:1 for example for spray dried chokeberry juice (Wilkowska et al. 2017). The next used proportion of 3:1 or 1:3 was used for spray drying of carrot juices (Janiszewska-Turak et al. 2017) or spray drying of barberry juice (Mahdavi et al. 2016).

With the spray drying process also temperature of evaporation is important, and that is related to the inlet temperature and feed flux rate (Janiszewska 2014; Tonon et al. 2009). This temperature is typically in the range of 140–240 °C, but for drying fruit and vegetable solutions it is recommended to use lower values i.e.140–180 °C (Janiszewska 2014; Tonon et al. 2009). This temperature can influence not only the quality and quantity of active ingredients but also the physical properties of powders such as water content, hygroscopicity, particle size etc. (Gawałek et al. 2017)

Jafari et al. (2017) testing pomegranate juice, Santhalakshmy et al. (2015) testing jamun juice, Tonon et al. (2011) testing acai berry juice and Gawałek et al. (2017) testing chokeberry juice noted, that the inlet temperature of drying, has an impact on the of carrier and physico-chemical properties of the powder and also on the quality and quantity of bioactive substances.

To our best knowledge there is limited research assessing the effects of spray drying chokeberry juice with different carrier proportions (MD and AG), saccharification of MD and drying inlet temperatures on the physical properties and active ingredients of obtained microcapsules of chokeberry juice. To improve the quality of the final product and extend information about properties of chokeberry powder the aim of the study was to examine the relationship of inlet air temperature and carrier type with amount of anthocyanins and polyphenols and selected physical properties of powders obtained after spray drying. The aims of the study are: (a) drying solutions with different carriers in the same conditions, (b) drying solutions with the same carrier at different temperatures, (c) testing selected physical and chemical properties of chokeberry juice powders, (d) testing selected physical and chemical properties after storage in different conditions. Moreover, the correlation between chosen physical properties with anthocyanin or polyphenol content was assessed.

## Materials and methods

### Materials

Chokeberry juice concentrate with (73.5°Bx) (Döhler company Sp. Z o.o., Mogielnica, Poland) was used for preparing solution for spray drying. Maltodextrine (MD10) with 10 DE (PPS “PEEPES” S.A., Łomża, Poland), maltodextrine (MD15) with 15.6 DE (PPS “PEEPES” S.A., Łomża, Poland) and arabic gum (AG) (Hortimex Sp. z o.o., Konin, Poland) were used as a carriers.

### Solutions for spray drying

Solution for spray drying was prepared from chokeberry concentrate (73°Bx). Concentrate was diluted to 12°Bx. To the juice carrierswere added. As a carrier material maltodextrine (MD10) with 10 DE, maltodextrine (MD15) with 15.6, arabic gum (AG) and their mixtures with different mass ratios (AG:MD10—1:1, 1:3, 3:1; AG:MD15—1:1, 1:3, 3:1) were used. To compare microcapsules from different carrier materials, soluble solid content in all the solutions was brought to 30°Bx by adding the appropriate amount of the carrier materials. The value of 30% of solid soluble content in solution was chosen on the basis of previous tests, in which the range 2:3 juice dry matter:carrier was the most suitable for spray drying. Obtained powders were not sticky to the dryer chamber and not dusty. The juice and carrier were mixed until all of the carrier was dissolved. The solutions containing arabic gum were left for 24 h in the fridge, because of it’s longer hydration time.

### Physical properties of solutions

The extract and the density of the chokeberry juice concentrate was examined, just as the extract, density and viscosity of it’s solutions made for the drying process. All measurements were replicated three times for each sample repetitions.

The soluble solids content (°Bx) was measured by digital refractometer PAL-3 (ATAGO, Japan).

The density (kg/m^3^) was measured by oscillating portable densitometer Densito 30PX (Mettler Toledo, Switzerland).

The apparent viscosity of solutions (mPa s) was measured by rotary viscometer (Brookfield, model DV-III, USA). The ULA (Ultra Low Adapter) spindle was used, with shear rate from 20 to 500 rpm. Sixteen ml of sample was used for testing. All of the measurements were performed at room temperature.

### Spray drying process

Drying was carried out in a semi-industrial spray dryer LAB S1 (Anhydro, Denmark). The inlet air temperature was 160 °C or 200 °C, used based on the previous tests and literature (Janiszewska 2014; Tonon et al. 2009). The disc speed, speed of raw material feed flux and air flow were 36,000 rpm, 0.6 × 10^−3^ m^3^/s and 0.055 m^3^/s, respectively. The outlet temperature was dependable on the inlet temperature, and ranged from 80 °C at 160 °C, to about 100 °C at 200 °C. The obtained powders were gathered in cyclone and moved into barrier opaque foil bags, welded by handheld welder. The drying trials were repeated two times for each experiment.

### Physical properties of powders

#### Dry matter

The dry matter (DM) was measured using oven-drying method. About 1 g of powder was measured and put into weighting dishes. The samples were dried at 105 °C temperature. Drying was performed until constant weight (about 4 h) (Janiszewska 2014). The amount of dry matter was calculated as the difference between the masses before and after drying. DM was measured in triplicate for each dried sample.

#### Water activity

The water activity was measured by Rotronic Hygroscop DT (Switzerland) using cylindrical probe Rotronic at temperature 25 °C. Obtained powder was placed into the plastic dish, and kept inside the rotronic until obtained constant result. Water activity was measured three times for each powder.

#### Colour

The colour components were measured on Minolta CR-A70 colorimeter (New Jersey, USA). CIE Lab 1976 color system was used as was described by Pieczykolan and Kurek (2019).For determination of colour components L* (lightness/darkness), a* (redness–greenness) and b* (blueness–yellowness). Calibration was made for a white pattern (L* 92.49, a* 1.25, b* − 1.92). The measurement was done on glass transparent petri dish on which the powder/juice was placed at 5 mm layer, standard illumination C, illuminant D65, angle for observation 2^o^ was used. The measurements were repeated five times for each powder.

#### Particles morphology and size

To determine the morphology of the particles, a scanning electron microscope Hitachi TM3000 (Hitachi, Japan) was used. Powder was placed on a two-sided sticky tape. The photos were taken by build-in navigation optical camera. The accelerating voltage was 5 kV.

The size of the particles was measured in particle size analyzer CILAS 1190 (France) by laser diffraction method. The measurement was performed in ethyl alcohol dispersion. The measurements were repeated ten times with 10–12% obscuration. The diameter of particles was expressed as d_50_.

#### Apparent density

The apparent density of particles was measured by helium pycnometer Sterepycnometer (Quantachrome Instruments, USA) as was presented by Domian et al. (2014). Calculation of apparent density was done in computer program dedicated to the pycnometer. The measurements were repeated three times.

#### Hygroscopicity

About 1 g of powder was weighted into an aluminum dish. The samples were placed into desiccators containing CH_3_COOK, K_2_CO_3_, MgCl_2_ and NaCl. Water activity of salt was 0.225, 0.4, 0.6 and 0.75, respectively. The samples were kept at room temperature. The mass was measured after 1, 2, 3, 24, 36, 48, 72 and 144 h for all samples. The alterations in the hygroscopicity properties of powder were described by a kinetic equation given by Janiszewska-Turak et al. (2017):

1 $$\frac{{m_{{{\text{H}}_{2} {\text{O}}}} }}{{m_{d.m.} }} = a + b\left( { 1 - \frac{1}{1 + bc\tau }} \right)$$

where a, b, and c are constant coefficients of equation; *τ* is time [h]; $m_{{{\text{H}}_{2} {\text{O}}}}$ is water gain in sample [g]; *m*_*d*.*m*._ is dry mass weight [g].

### The total monomeric anthocyanin concentration

The total monomeric anthocyanin concentration was analyzed by the pH differential method according to Giusti and Wrolstad (2000). Extract of chokeberry juice, solutions and powders were diluted with two buffer solutions at pH 1 and pH 4.5. Cy-3-G (MW = 449.2 g/mol), used as a standard. To 0.6 g ± 0.001 g of spray-dried powder 30 ml of distilled water was added, then vortexed till obtained homogeneous solution. Prepared material was infiltrated into volumetric flask and filled to 50 ml with distilled water (Nowacka et al. 2014). Two test-tubes for each sample were prepared. 3.5 ml of buffer with pH = 1 (0.025 M KCl) was poured into first test-tube, and 3.5 ml of buffer with pH = 4.5 (0.4 M CH_3_COONa) into the second one. 1.5 ml of water extract was added to each test-tube, and then they were vortexed once more. The test-tubes with content was left for 30 min in dark place at room temperature, and then absorbance was measured. Results were expressed as mg of Cy-3-G per L (Horszwald et al. 2013). The absorbance was measured with wave length 450 nm, 510 nm and 700 nm, in relation to buffers with pH = 1 and pH = 4.5. The measurements were done at spectrophotometer Thermo Spectronic Helios Gamma (Thermo Fischer Scientific, USA). There were three repetition for each material.

### Polyphenols content (TPC)

The extracts were prepared according to methodology presented by Nowacka et al. (2014) with some modifications. Water extracts were prepared for measurement: spray-dried powders (A), chokeberry juice concentrate (B) and solution of chokeberry juice (C). For this purpose 0.3 g of material A, 0.2 g of material B and 0.8 g of material C (± 0.0001 g precision) were weighted into beaker, and then approximately 30 ml of distilled water was added. The samples were mixed. The result content was infiltrated into volumetric flasks and filled to 50 ml with distilled water. Polyphenol content was measured spectrophotometrically according to Folin–Ciocalteau’s method (van der Sluis et al. 2002). The absorbance was measured in spectrophotometer Thermo Spectronic Helios Gamma (Thermo Fischer Scientific, USA), with wave length 750 nm, in relation to the sample without extract. The measurements were repeated two times.

### Storage

All powders were stored for 2 months at two temperatures, 4 and 25 °C, in a dark place. During storage glass transition phenomenon did not take place [Tg as was mentioned in literature for chokeberry powders was higher than 65 °C (Pieczykolan and Kurek 2019)]. After that time, colour and monomeric anthocyanins and polyphenols were tested as described in the methodology.

### Statistical methods

Data are presented as mean ± standard deviation. Significance of inter-group differences was determined by one-way analysis of variance (ANOVA), calculated with Statistica v. 13.3 software (StatSoft Poland). Individual group differences were identified using the Tukey (HSD) multiple range test at a significance level of 0.05. Coefficients of determination (R^2^) and root mean square errors (RMSE) were used in this study to determine the goodness of fit. Fitting hygroscopicity to equations was performed using Table Curve 2D v 5.01 (SYSTAT Software Inc). Additionally, Pearson’s correlation coefficient was calculated in order to evaluate the dependence between anthocyanin content and colour parameters.

## Results and discussion

### Physical properties of chokeberry juice and its carrier solutions

Juice was characterize by soluble solid content 12°Bx, which was each time determined by diluting the juice concentrate. Viscosity of chokeberry juice was 3.4 ± 0.3 mPa s, density 1042 ± 4 kg/m^3^, polyphenol content 8888 ± 274 mg/100 g d.m., anthocyanin content 9329 ± 365 mg/100 g d.m.. Content of anthocyanin and polyphenols depends on the variety and method of cultivation and usually varies for anthocyanin in the range 6.2–6.7 g/kg in berries and juice 5–473 g/L (Mayer-Miebach et al. 2012; Wilkowska et al. 2017), and for polyphenol in juice approx. 651 + 25 GA/100 g. The colour coefficients for juice was L* 2.76 ± 0.10, a* 1.08 ± 0.25 b* 0.49 ± 0.21 which is related to almost black colour.

Creating juice with carrier solutions resulted in increase of the solid soluble content in the solution (from 12 to 30°Bx) and caused a significant increase in its density. Change of carrier material from MD to AG and from MD15 to its mixture with AG caused a statistically significant increase in density of solutions, whereas for MD10 this trend was not observed (Table 1).

**Table 1:** Physical and rheological properties of chokeberry juice solutions

Tested solution	Density (kg/m^3^) Xav ± SD	Viscosity (mPa s) Xav ± SD
MD10	1117 ± 1^b^	9.6 ± 0.1^ab^
MD15	1119 ± 0^bc^	5.6 ± 0.0^a^
AG:MD10 1:3	1116 ± 1^b^	18.3 ± 0.6^c^
AG:MD10 1:1	1122 ± 2^cd^	26.3 ± 0.1^d^
AG:MD10 3:1	1118 ± 1^b^	49.3 ± 0.6^e^
AG:MD15 1:3	1123 ± 3^d^	12.2 ± 0.0^b^
AG:MD15 1:1	1122 ± 0^d^	25.5 ± 0.1^d^
AG:MD15 3:1	1122 ± 1^cd^	29.3 ± 0.1^d^
AG	1123 ± 3^d^	56.3 ± 0.9^f^
Juice	1042 ± 4^a^	3.4 ± 0.1^a^

a–d values with different letters in the same column differ significantly (*p* < 0.05)

All of the tested solutions were Newtonian fluids. This is common for most of the juices from fruits and vegetables; usually carrot juices are exceptions (Janiszewska 2014; Janiszewska-Turak et al. 2017). Addition of carrier material significantly increases solutions’ viscosity, only because of the increase in solid soluble content. Moreover, it was noted that the carrier materials have a significant impact on apparent viscosity of the solutions. The solution with arabic gum as a carrier had the highest viscosity (56.3 ± 0.9 mPa s), and the one with maltodextrin 15DE as a carrier had the lowest viscosity (5.6 ± 0.0 mPa s) (Table 1). It was observed that replacement of MD with AG caused an increase in the viscosity values. Moreover, the increase was proportional to the concentration of arabic gum. Analyzing the influence of the higher saccharification level of maltodextrins, it was observed that an increase in dextrose equivalent caused a decrease in viscosity values (Table 1). The same results for maltodextrin solutions, without juices, were presented by Pycia et al. (2016). Arabic gum probably because of its emulsifying abilities and its larger mass particles can influence viscosity by increasing the value. The same results were obtained by Janiszewska-Turak et al. (2017) for carrot juice solutions with carriers and Janiszewska (2014) for beetroot juice solutions with carriers. A different relationship was obtained by Tonon et al. (2008) for acai juice: an increase in maltodextrin concentration also caused increased viscosity.

### Selected physical properties of powders

The amount of dry matter in obtained powders varied from 97.1% for chokeberry juice with AG:MD10 3:1 dried at 160 °C to 99.1% for chokeberry juice with AG:MD15 1:1 dried at 200 °C (Table 2). According to Clarke (2003) the content of dry matter in powders should be higher than 95%, because high dry matter content can extend the powder’s usefulness for technological purposes and increase stability of their physical properties. The present results indicated that obtained chokeberry powders had an appropriate dry matter content. Similar values of dry matter for chokeberry powders were obtained by Pieczykolan and Kurek (2019). No significant influence of temperature, carrier type or saccharification level was observed. Different results were obtained by Goula and Adamopoulos (2008) examining the impact of saccharification level of maltodextrin on tomato powder properties. They noted that the higher the DE of maltodextrin was, the lower was dry matter in obtained powders. They suggested that it could be connected to maltodextrin’s chemical structure, which is attributed to the fact that the lower the dextrose equivalent (DE) of a MD is, the higher the glass transition temperature and it leads to a slower evaporation rate due to the higher resistance to mass transfer.

**Table 2:** Selected physical properties of powders

Tested powder	Dry matter (%) Xav ± SD	Water activity (–) Xav ± SD	Apparent density (kg/m^3^) Xav ± SD	d_50_ (µm) Xav ± SD	L* (–) Xav ± SD	L* after storage 4 °C (–) Xav ± SD	L* after storage 25 °C (–) Xav ± SD
MD10 160 °C	98.4 ± 0.03^a^	0.212 ± 0.014^cd^	1166 ± 3.5^h^	29.6 ± 2.3^a–e^	46.2 ± 0.2^a–cA^	47.8 ± 0.9^a–cAB^	48.1 ± 0.5^a–dC^
MD10 200 °C	98.7 ± 0.09^a^	0.202 ± 0.013^b–d^	1091 ± 2.9^f^	37.7 ± 2.6^e^	46.0 ± 0.2^abA^	48.8 ± 0.5^bcB^	48.3 ± 0.3^a–dB^
MD15 160 °C	98.6 ± 0.31^a^	0.234 ± 0.004^de^	1130 ± 2.6^g^	33.3 ± 2.0^d–f^	45.8 ± 0.2^abA^	47.2 ± 0.3^a–cB^	47.1 ± 0.4^a–dB^
MD15 200 °C	97.9 ± 0.01^a^	0.229 ± 0.006^de^	1181 ± 5.0^i^	28.1 ± 2.1^a–c^	45.8 ± 0.1^abA^	48.4 ± 0.1^a–cB^	49.2 ± 1.3^b–dB^
AG:MD10 1:3 160 °C	98.1 ± 0.04^a^	0.206 ± 0.005^b–d^	1221 ± 1.4^l^	32.8 ± 0.6^c–f^	45.3 ± 0.3^aA^	48.3 ± 0.1^a–cC^	46.4 ± 0.2^abB^
AG:MD10 1:3 200 °C	97.8 ± 0.49^a^	0.185 ± 0.018^a–c^	1053 ± 4.2^e^	32.6 ± 1.1^c–e^	46.7 ± 0.9^a–cA^	47.8 ± 0.5^a–cA^	47.3 ± 0.7^a–dA^
AG:MD10 1:1 160 °C	98.0 ± 0.03^a^	0.211 ± 0.001^cd^	1217 ± 0.8^kl^	29.1 ± 0.9^a–e^	46.0 ± 0.3^abA^	47.4 ± 0.4^a–cB^	48.2 ± 0.2^a–dB^
AG:MD10 1:1 200 °C	98.3 ± 0.15^a^	0.174 ± 0.002^ab^	1059 ± 0.7^e^	33.6 ± 0.9^d–f^	46.0 ± 0.7^abA^	47.5 ± 0.1^a–cA^	47.0 ± 1.8^a–dA^
AG:MD10 3:1 160 °C	97.1 ± 0.001^a^	0.186 ± 0.003^a–c^	1279 ± 2.4^n^	27.5 ± 0.4^ab^	47.1 ± 1.1^a–cA^	47.6 ± 0.5^a–cA^	49.4 ± 1.4^cdA^
AG:MD10 3:1 200 °C	98.6 ± 0.15^a^	0.158 ± 0.013^a^	995 ± 0.8^b^	34.1 ± 0.6^ef^	45.9 ± 0.8^abA^	46.7 ± 0.5^aA^	47.9 ± 0.5^a–dA^
AG:MD15 1:3 160 °C	97.4 ± 0.01^a^	0.257 ± 0.013^e^	1208 ± 5.5^jk^	24.8 ± 0.9^a^	46.8 ± 0.7^a–cA^	47.1 ± 0.5^abA^	48.1 ± 0.5^a–dA^
AG:MD15 1:3 200 °C	98.4 ± 0.03^a^	0.202 ± 0.004^b–d^	1172 ± 1.9^hi^	32.1 ± 1.5^b–e^	47.9 ± 0.3^bcB^	48.6 ± 0.2^a–cB^	46.8 ± 0.3^a–cA^
AG:MD15 1:1 160 °C	97.9 ± 0.09^a^	0.169 ± 0.003^a^	1198 ± 5.4^j^	28.8 ± 0.5^a–d^	48.2 ± 0.9^cA^	47.0 ± 0.2^abA^	46.0 ± 0.5^aA^
AG:MD15 1:1 200 °C	99.1 ± 0.25^a^	0.215 ± 0.004^cd^	1033 ± 3.9^d^	47.7 ± 5.6^g^	46.8 ± 1.0^a–cA^	47.9 ± 0.2^a–cA^	46.0 ± 0.5^aA^
AG:MD15 3:1 160 °C	97.8 ± 0.08^a^	0.159 ± 0.000^a^	1244 ± 5.0^m^	28.9 ± 0.8^a–d^	47.6 ± 0.5^bcA^	47.6 ± 0.4^a–cA^	49.2 ± 0.7^b–dA^
AG:MD15 3:1 200 °C	97.8 ± 0.55^a^	0.175 ± 0.001^ab^	1022 ± 3.2^c^	32.8 ± 0.6^c–f^	46.1 ± 1.3^a–cA^	48.2 ± 0.5^a–cA^	48.4 ± 0.5^a–dA^
AG 160 °C	98.0 ± 0.07^a^	0.174 ± 0.004^ab^	1235 ± 4.9^m^	29.1 ± 0.4^a–e^	45.5 ± 0.5^aA^	47.3 ± 1.0^a–cA^	47.4 ± 0.2^a–dA^
AG 200 °C	97.8 ± 0.17^a^	0.165 ± 0.001^a^	970 ± 2.2^a^	33.2 ± 0.3^d–f^	46.5 ± 0.3^a–cA^	49.0 ± 0.1^a–cB^	49.8 ± 0.7^dB^

Tested powder	a* (–) Xav ± SD	a* after storage 4 °C (–) Xav ± SD	a* after storage 25 °C (–) Xav ± SD	b* (–) Xav ± SD	b* after storage 4 °C (–) Xav ± SD	b* after storage 25 °C (–) Xav ± SD
MD10 160 °C	37.4 ± 0.28^jA^	36.8 ± 1.24^AG^	36.4 ± 0.19^AG^	1.13 ± 0.07^hA^	0.99 ± 0.41^e–AG^	0.96 ± 0.16^bcA^
MD10 200 °C	37.1 ± 0.19^jB^	34.4 ± 0.52^d–AG^	35.1 ± 0.16^fAG^	1.07 ± 0.09^hA^	0.70 ± 0.30^d–fA^	0.96 ± 0.27^bcA^
MD15 160 °C	36.2 ± 0.79^ijA^	36.3 ± 0.11^fAG^	34.9 ± 0.46^fAG^	1.02 ± 0.19^ghA^	2.04 ± 0.15^gB^	1.04 ± 0.09^cA^
MD15 200 °C	37.5 ± 0.22^jC^	35.7 ± 0.33^fgB^	33.6 ± 0.52^c–fA^	1.42 ± 0.08^hA^	1.21 ± 0.22^fAG^	1.00 ± 0.70^cA^
AG:MD10 1:3 160 °C	36.4 ± 0.25^ijB^	34.0 ± 0.17^c–fA^	36.3 ± 0.19^gB^	0.57 ± 0.07^fAG^	0.45 ± 0.09^c–fA^	0.65 ± 0.11^a–cA^
AG:MD10 1:3 200 °C	35.1 ± 0.86^hiA^	34.3 ± 0.34^c–fA^	34.5 ± 0.11^d–AG^	0.34 ± 0.14^efA^	0.66 ± 0.08^d–fA^	0.36 ± 0.20^a–cA^
AG:MD10 1:1 160 °C	35.0 ± 0.07^hiC^	33.1 ± 0.01^b–eB^	32.4 ± 0.01^cdA^	0.00 ± 0.09^deA^	0.14 ± 0.02^b–fA^	− 0.02 ± 0.04^a–cA^
AG:MD10 1:1 200 °C	33.9 ± 0.05^ghB^	32.6 ± 0.11^b–dA^	32.7 ± 0.71^c–eAB^	− 0.05 ± 0.09^deA^	− 0.13 ± 0.04^b–eA^	0.46 ± 1.03^a–cA^
AG:MD10 3:1 160 °C	30.2 ± 0.31^a–cA^	31.9 ± 0.71^bcAB^	32.1 ± 0.62^bcB^	− 0.86 ± 0.23^bA^	− 0.69 ± 0.35^a–cA^	− 0.07 ± 0.17^a–cA^
AG:MD10 3:1 200 °C	30.4 ± 0.59^a–cA^	31.4 ± 0.57^abA^	30.1 ± 0.00^abA^	− 0.98 ± 0.17^abA^	− 0.91 ± 0.23^abA^	− 0.95 ± 0.01^aA^
AG:MD15 1:3 160 °C	33.0 ± 0.50^fAG^	35.3 ± 0.21^e–gB^	33.8 ± 0.23^c–fA^	0.30 ± 0.16^efA^	0.49 ± 0.10^c–fA^	0.26 ± 0.18^a–cA^
AG:MD15 1:3 200 °C	31.4 ± 0.57^c–eA^	34.0 ± 1.32^c–fAB^	34.8 ± 0.30^e–gB^	− 0.11 ± 0.15^deA^	0.75 ± 0.75^d–fA^	0.56 ± 0.05^a–cA^
AG:MD15 1:1 160 °C	29.9 ± 0.29^abA^	33.2 ± 0.42^b–eB^	34.2 ± 0.16^c–gB^	− 0.79 ± 0.20^bcA^	− 0.17 ± 0.33^b–eA^	− 0.12 ± 0.21^a–cA^
AG:MD15 1:1 200 °C	32.3 ± 0.80^efAB^	30.8 ± 0.13^abA^	33.4 ± 0.05^c–fB^	− 0.37 ± 0.24^cdA^	− 0.37 ± 0.16^a–dA^	− 0.15 ± 0.13^a–cA^
AG:MD15 3:1 160 °C	32.1 ± 0.14^d–fB^	32.6 ± 1.10^b–dB^	30.0 ± 0.21^abA^	− 1.03 ± 0.09^abA^	− 0.59 ± 0.53^a–cA^	− 0.84 ± 0.25^aA^
AG:MD15 3:1 200 °C	30.7 ± 0.41^b–dA^	30.8 ± 0.30^abA^	32.4 ± 0.02^cdB^	− 0.70 ± 0.28^bcAB^	− 0.96 ± 0.05^abA^	− 0.18 ± 0.16^a–cB^
AG 160 °C	30.1 ± 0.56^a–cA^	31.1 ± 0.64^abAB^	32.5 ± 0.77^cdB^	− 1.10 ± 0.13^abA^	− 0.98 ± 0.32^abA^	− 0.05 ± 0.69^a–cA^
AG 200 °C	29.1 ± 0.09^aA^	29.1 ± 0.35^aA^	29.6 ± 1.70^aA^	− 1.38 ± 0.06^aA^	− 1.48 ± 0.10^aA^	− 0.80 ± 1.06^abA^

a–d values with different letters in the same column differ significantly (*p* < 0.05)

A–D values with different letters denoted in rows for the same parameter before and after storage differ significantly (*p* < 0.05)

The next parameter with which we can predict powders’ behaviour under storage is water activity. Bacteria, yeasts and moulds grow in an environment with water activity above 0.6. Resultant water activity of powders (below 0.3) not only provides microbiological stability, but also reduces enzymatic activity, lipid oxidation and caking (Janiszewska-Turak et al. 2017; Labuza and Altunakar 2007). All obtained powders have water activities below 0.26. The highest water activity (0.257) was recorded for the powder containing AG:MD15 1:3 as a carrier, dried at 160 °C (Table 2). In most cases water activity for powders dried at 200 °C was lower than for the ones dried at 160 °C, exception were powders AG:MD 1:1 and AG:MD 3:1, which is connected with faster water evaporation. It was found that the greater the addition of arabic gum, the lower was the water activity in powders. Janiszewska-Turak et al. (2017) came to the same conclusions when carrots juices were dried with carrier material.

**Fig. 1:**
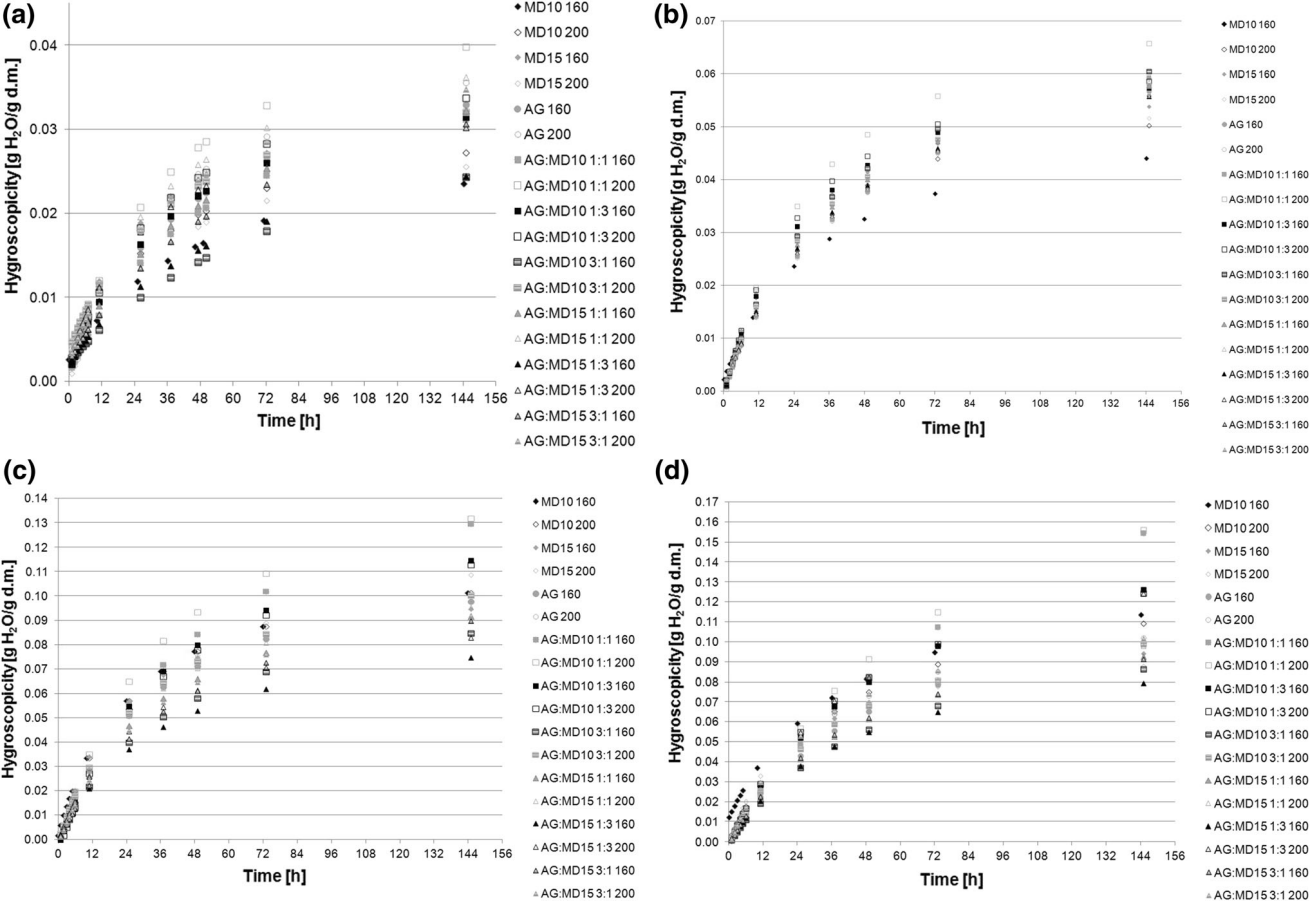
Hygroscopicity of chokeberry powders at: **a** a_w_ = 0.225, **b** a_w_ = 0.4, **c** a_w_ = 0.6, **d** a_w_ = 0.75

Hygroscopicity is a parameter based on which it is possible to predict powder behaviour under storage, and show stability of powders. In the present research the lowest water absorbance was recorded for the environment with water activity at 0.225, and the highest for the environment with water activity at 0.75 (Fig. 1). The highest amount of water was absorbed by the powder containing AG:MD10 1:1 dried at 200 °C, regardless of storage environment. It has absorbed water from 0.04 to 0.16 g H_2_O/g d.m. in 144 h (Fig. 1). In the environment with water activity at 0.225 and 0.4 the smallest amount of water was absorbed by powder containing maltodextrin with saccharification level 10DE, dried t 160 °C. In environment with water activity at 0.6 and 0.75 the powder dried at 160 °C containing AG:MD15 1:3 has the lowest values of absorbed water, 0.02–0.04 g H_2_O/g d.m. and 0.08 g H_2_O/g d.m. in 144 h, respectively (Fig. 1). In environments with water activity below 0.5 was noticed that concentration of gum arabic had an impact on the amount of water absorbed by powders—the absorption was higher [similar results were obtained by Janiszewska (2014) and da Silva Carvalho et al. (2016)], and in environments with water activity above 0.5 no impact of carrier type on powders hygroscopicity was observed. The powders created at higher temperature were more hygroscopic, which is related to the size of particle cramp. A similar relationship was observed by Santhalakshmy et al. (2015) when drying jamun fruit juice. No differences in course of absorption kinetic curves were noted. The powders absorbed a larger amount of water during the first 24 h. Using kinetic equations, the equilibrium constants for relative increase of water mass were determined. None of the powders reached a plateau, although the powders stored in the environment with water activity at 0.75 were closest to reach that state (Table S1). Diffusive properties have an impact on food ingredient content, because the greater the ability to absorb water, the greater is the water content in material and the faster the biochemical processes, causing valuable ingredients loss.

All of the powders had values of the RMSE model, below 1%. The coefficient of determination was high for all of the powders stored in different water activity environments. The value of the coefficient was from 0.95 to 1. The highest value of the coefficient of determination was recorded in environments with water activity at 0.6 and 0.75, and the lowest at 0.225 and 0.4 (Table S1). Those results show a perfect fit obtained data to the chosen model, which can gave us an opportunity to calculate with good precision the absorption rate.

There were observed differences in apparent density of obtained chokeberry juice powders. The powder dried t 160 °C containing AG:MD15 3:1 as a carrier had the highest apparent density, and the powder dried at 200 °C containing only arabic gum had the lowest (Table 2). The inlet air temperature had a statistically significant impact on apparent density of powders; the higher the temperature, the lower the apparent density, with the exception of powders based on MD15. Jafari et al. (2017) obtained the same result during testing pomegranate juices. The reason for this might be the acceleration of water vaporization at higher temperatures, causing faster shell creation and bigger particles (confirmed in our research—Table 2). It was also noted that the percentage content of arabic gum had an impact on apparent density of chokeberry powders. The powders obtained at 160 °C increased the apparent density when the percentage content of AG was increasing, but at 200 °C the relationship was inverted; the higher the AG content, the lower the apparent density. A similar relationship was observed by Tonon et al. (2010) for acai berry juice. It might be caused by lower density of arabic gum compared to powders based on maltodextrin.

**Fig. 2:**
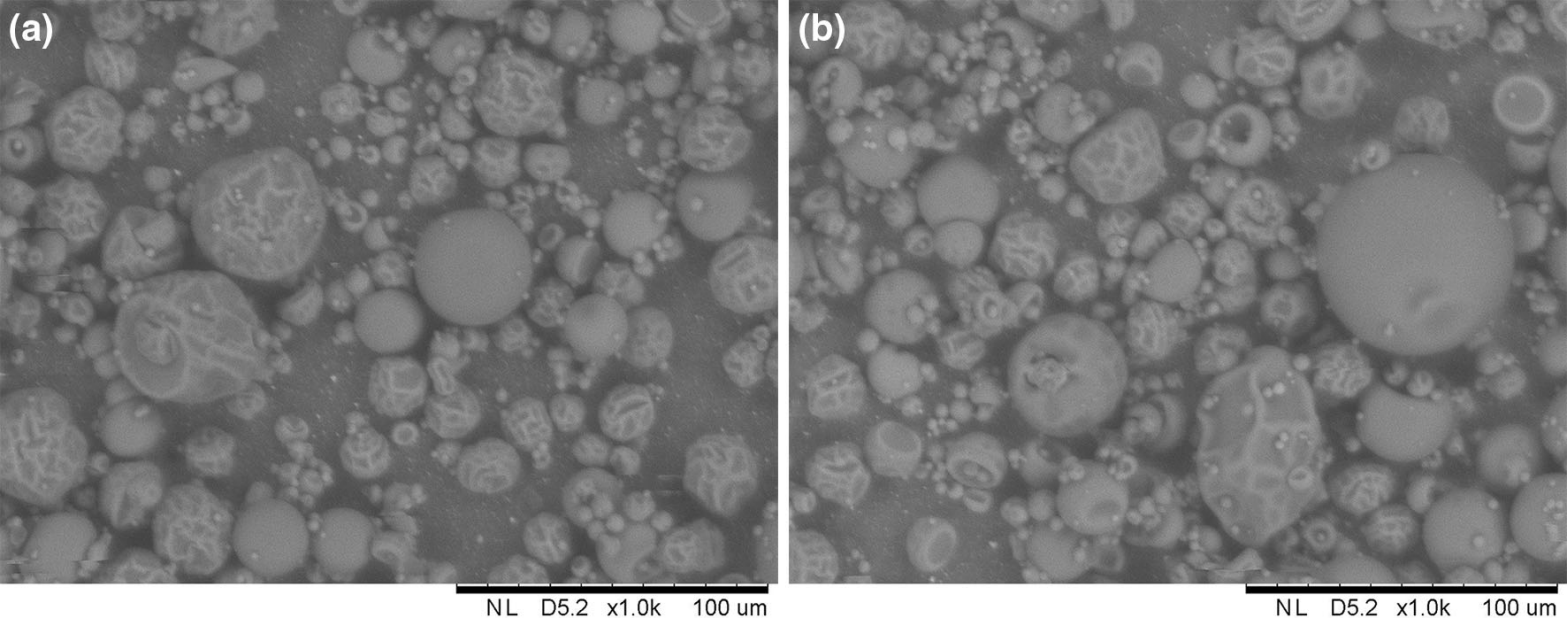
Chokeberry juice powder morphology for powder based on MD10 dried at **a** 160 °C, **b** 200 °C (magnification ×1000, bar length 100 µm)

Obtained chokeberry particles had similar size, and their diameter values were between 24.8 ± 0.9 µm (AG:MD15 1:3 160 °C) and 37.7 ± 2.6 µm (MD10 200 °C) (Table 2). The only exception was the powder containing AG:MD15 1:1, dried at 200 °C whose particles had a bigger diameter 47.7 ± 5.6 µm. In the literature it can be found that inlet temperature increase resulted in larger diameter of particles. In our data we have observed the same tendency (exception were powders based on MD15), but the increase was not statistically significant for some of our powders (Table 2). Jafari et al. (2017), Pieczykolan and Kurek (2019), Santhalakshmy et al. (2015), Tonon et al. (2008) have reported a similar tendency with increasing temperature as in our research. Moreover, the shape of particle impacted their size, and the particles created at lower temperature were more ridged and had bigger cracks in (Fig. 2). The carrier material had no statistically significant impact on particles’ diameter (Table 2).

Increase in DE value from 10 to 15 did not cause statistically significant effect on the diameter size; exceptions were powders dried at 200 °C for MD (decrease) and AG:MD = 1:1 (increase). The same result was obtained by Gawałek et al. (2017) for spray dried chokeberry juice; they did not see any significant effect with increasing dextrose equivalent from 8 to 18.

It was observed that most of the particles had a creased, spherical shape, which is common for spray dried products. Because of that, only chosen pictures from SEM are shown with powders based on MD10 (Fig. 2a, b). Some of the particles were damaged. It was observed that in products dried at higher temperatures there were more particles with a smooth surface than in products dried t 160 °C, but also there were more damaged ones (Fig. 2). The same conclusions were reached by Jafari et al. (2017) during pomegranate juice drying. It was caused by the fact that during faster water vaporization a harder, more rigid and smoother particle is created. Drying at lower temperatures makes the particle wetter and moreflexible, and it collapses (Jafari et al. 2017; Wilkowska et al. 2017). No impact of carrier type or saccharification on particle morphology was observed. Similar results for saccharification level were observed by Gawalek et al. (2017) after analyzising chokeberry juice particles via spray drying. Nevertheless, Jafari et al. (2017) during examination of pomegranate juice reached the conclusion that higher concentration of maltodextrin as a carrier causes more particle ridging, because of its ability to migrate in the outer zones of particle, reducing its durability, which is confirmed in tests performed by Tonon et al. (2008).

Comparing colour of the juice to the colour of powders it is observed that powders are lighter than juice itself, but b* values for juice and powders were similar.

Analysis of the powder colour results showed that drying temperature had no significant impact on measured colour parameters. The exceptions were parameter a* for powders based on AGMD15 1:1 and AGMD15 1:3, and parameter b* for powder AGMD 1:3. There was no statistically significant impact of carrier type on the colour of obtained powders, and there was also no unambiguous trend when comparing different percentage contenta of carriers type (e.g. MD and its mixture with AG). The powder containing AGMD15 1:1 (160 °C) had the highest lightness (L*)-, and AGMD10 1:3 (160 °C) has been described by the lowest lightness values (Table 2). For all of the powders the colour parameter a* had positive values (Table 2). It was observed that carrier type had a statistically significant impact on colour parameters a* and b*. With the increase of arabic gum content in carrier solution, it was observed that examined colour parameters were decreasing, regardless of the saccharification level of maltodextrin. The same conclusions were reached by Janiszewska (2014) when examining powders from beetroot juice. An opposite relationship was described by Dłużewska et al. (2011) when testing β-carotene concentrate added to the carrier mixed with water. The differences in the described research could be related to the preparation of solutions for spray drying, and also the active ingredient itself which has different colour coefficients than in chokeberry juice.

Storage had a statistically significant impact on the colour parameter L* value in the powders based on MD10, MD15, AG:MD10 1:3 160 °C, AG:MD10 1:1 160 °C and AG 200 °C. The increase of this parameter occurred for both storage temperatures. Moreover, the powders stored in 25 °C had higher L* parameter values. Similar results were obtained by Moser et al. (2017) testing dried grape juice, stored for 150 days t 35 °C.

The temperature of drying and the carrier type had no significant impact on L* parameter values during storage at both used temperatures.

Statistically significant changes in colour parameter a* value were observed during chokeberry juice powders storage. It might be caused by decreasing the amount of colourants that stabilize red colour, belonging to anthocyanins (Lago-Vanzela et al. 2014). A similar relationship was recorded by Oezen et al. (2011) when testing changes during storage of Turkish delight, in which black carrot juice was used as a colourant. The opposite effect was observed by Moser et al. (2017) storing grape juice powder; the a* parameter value increased after 5 months of storage. Comparing values for both storage temperatures no clear trend was observed. There was no statistically significant impact of drying temperature on a* parameter value during storage in both temperatures. The exceptions were powders based on AG:MD15 3:1 and AG stored at 25 °C. A statistically significant impact of carrier type on colour parameter the a* was observed for storage at both temperatures. This value decreased when the concentration of arabic gum increased. The opposite relationship was observed by Dłużewska et al. (2011) who stored β-carotene microencapsulated by spray drying.

A statistically significant change during storage was recorded only for powders based on MD15 160 °C and AG:MD15 3:1 200 °C. No statistically significant impact of drying temperature on this parameter value during storage in 4 °C and 25 °C was observed. The carrier type had no statistically significant impact on powders stored in 25 °C, but at a lower temperature powders with a higher amount of arabic gum differed significantly from powders with a higher amount of maltodextrin. Increase in concentration of arabic gum caused a decrease in colour parameter b*.

### Anthocyanins content

As mentioned in point 3.1 the anthocyanins content in juice was 9329 ± 365 mg/100 g d.m.. Each time a rapid decrease in anthocyanin content was observed after spray drying., The anthocyanin content in obtained powders ranged from 1694 to 2028 mg/100 g (Table 3). This value for powders is lower than the value obtained by Horszwald et al. (2013) after spray of chokeberry juice drying in 180 °C—4800 mg/100 g, but similar to the value obtained for chokeberry powders based on MD:AG presented by Pieczykolan and Kurek (2019) (1940 mg/100 g). The highest content of anthocyanins was recorded for powder based on AG:MD10 1:3 (160 °C), and the lowest for powder based on AG:MD15 1:1 (200 °C). Statistical analysis showed that carrier type had no significant impact on anthocyanin content. However, with the increase of maltodextrin saccharification level a decrease of anthocyanin content in obtained powders was observed. Similar results were obtained by Jafari et al. (2017) for dried pomegranate juice, Bakowska-Barczak and Kolodziejczyk (2011) for blackcurrant powder and Tonon et al. (2010) for acai juice powder. Moreover, Tonon et al. (2008) and Bakowska-Barczak and Kolodziejczyk (2011) observed that drying at higher temperature causes higher anthocyanins degradation. The reason was their high sensitivity to high temperatures (Jafari et al. 2017).

**Table 3:** Content of selected phenol compounds in chokeberry powders

Tested powder	Anthocyanin (mg/100 g d.m) Xav ± SD	Anthocyanin after storage 4 °C (mg/100 g d.m.) Xav ± SD	Anthocyanin after storage 25 °C (mg/100 g d.m.) Xav ± SD	Polyphenols (mg/100 g d.m.) Xav ± SD	Polyphenols after storage 4 °C (mg/100 g d.m.) Xav ± SD	Polyphenols after storage 25 °C (mg/100 g d.m.) Xav ± SD
MD10 160 °C	2002 ± 19^bcA^	2124 ± 41^cB^	1899 ± 10^abA^	2475 ± 88^a–cA^	2969 ± 104^bB^	2954 ± 105^aB^
MD10 200 °C	1957 ± 40^bcA^	1967 ± 125^a–cA^	1815 ± 60^abA^	2438 ± 200^abA^	3512 ± 81^aB^	2111 ± 205^aA^
MD15 160 °C	1830 ± 106^a–cA^	1856 ± 5^aA^	1734 ± 51^abA^	2332 ± 21^aA^	3085 ± 115^abAB^	2737 ± 219^aB^
MD15 200 °C	1774 ± 109^abA^	1959 ± 54^a–cA^	1785 ± 104^abA^	2340 ± 94^aA^	2989 ± 120^abA^	2734 ± 263^aA^
AG:MD10 1:3 160 °C	2028 ± 50^cA^	2086 ± 40^bcA^	1855 ± 125^abA^	3154 ± 238^b–dA^	3173 ± 227^bA^	3004 ± 171^aA^
AG:MD10 1:3 200 °C	2020 ± 83^bcA^	1925 ± 12^a–cA^	1863 ± 48^abA^	3206 ± 10^b–dA^	3219 ± 227^abA^	2770 ± 263^aA^
AG:MD10 1:1 160 °C	1878 ± 36^a–cA^	2019 ± 52^a–cA^	1905 ± 76^abA^	3152 ± 39b^cdA^	3198 ± 330^abA^	2699 ± 138^aA^
AG:MD10 1:1 200 °C	1958 ± 77^bcA^	2079 ± 35^bcA^	1727 ± 137^abA^	3225 ± 86^cdA^	3069 ± 311^bA^	2870 ± 190^aA^
AG:MD10 3:1 160 °C	1889 ± 138^a–cA^	1991 ± 7^a–cA^	1601 ± 150^aA^	3386 ± 346^dA^	3197 ± 103^bA^	3059 ± 53^aA^
AG:MD10 3:1 200 °C	1945 ± 28^a–cA^	1976 ± 6^a–cA^	1949 ± 12^bA^	3478 ± 425^dA^	3367 ± 260^abA^	2730 ± 173^aA^
AG:MD15 1:3 160 °C	1927 ± 9^a–cA^	2077 ± 31^bcB^	1885 ± 40^abA^	3227 ± 293^cdA^	3131 ± 101^abA^	2541 ± 236^aA^
AG:MD15 1:3 200 °C	1873 ± 5^a–cA^	1888 ± 60^abA^	1799 ± 95^abA^	3080 ± 172^a–dA^	3256 ± 246^abA^	2634 ± 276^aA^
AG:MD15 1:1 160 °C	1844 ± 15^a–cA^	2007 ± 28^a–cB^	1869 ± 17^abA^	3022 ± 64^a–dA^	3231 ± 122^bB^	3106 ± 34^aAB^
AG:MD15 1:1 200 °C	1695 ± 39^aA^	1896 ± 25^abB^	1750 ± 32^abA^	3103 ± 16^a–dA^	3256 ± 34^bA^	2998 ± 127^aA^
AG:MD15 3:1 160 °C	1884 ± 58^a–cA^	2027 ± 57^a–cA^	1874 ± 40^abA^	2912 ± 186^a–dA^	3238 ± 82^bA^	3085 ± 185^aA^
AG:MD15 3:1 200 °C	1901 ± 38^a–cAB^	2000 ± 63^a–cB^	1756 ± 1^abA^	3673 ± 113^dB^	3548 ± 42^bAB^	2999 ± 95^aA^
AG 160 °C	1936 ± 49^a–cA^	1990 ± 93^a–cA^	1795 ± 107^abA^	3109 ± 200^a–dA^	3340 ± 230^bA^	2962 ± 24^aA^
AG 200 °C	1844 ± 21^a–cAB^	1897 ± 2^abB^	1744 ± 38^abA^	3465 ± 110^dA^	3334 ± 178^bA^	3121 ± 29^aA^

a–d values with different letters in the same column differ significantly (*p* < 0.05)

A–D values with different letters denoted in rows for the same parameter before and after storage differ significantly (*p* < 0.05)

For the storage temperature of powders it was observed, that storage at 4 °C caused an increasing trend of anthocyanins content (with the exception of powder based on AG:MD10 1:3 dried in 200 °C); however analyzing anthocyjanin content in powder storage at 25 °C a decreasing trend was observed (with the exception of five powders: MD15 200 °C, AG:MD10 1:1 160 °C, AG:MD10 3:1 200 °C, AG:MD15 1:1 160 °C and AG:MD15 1:1 200 °C) (Table 3). However the observed trends were statistically significant only for powders based on mixture MD15 with AG storage st 4 °C. Similar trends were observed by Bakowska-Barczak and Kolodziejczyk (2011) who stored for 3 months powders from blackcurrant obtained with maltodextrins with different saccharification levels, and by Jiménez-Aguilar et al. (2011) who stored for month powders from blueberry, obtained with mesquite gum. In they research anthocyanins content increased during storage at 4 °C and 8 °C, but during storage at 25 °C the stability of these compounds decreased.

The highest anthocyanin content during storage at 4 °C was recorded for powder based on MD10 dried at 160 °C, and during storage at 25 °C for powder based on AG:MD10 3:1 dried in 200 °C. The lowest anthocyanins content during storage at 4 °C and 25 °C were recorded respectively for powders based on MD15 160 °C and AG:MD10 3:1 160 °C (Table 3). The carrier type and drying temperature had no statistically significant impact on anthocyanins content in powders stored at 4 °C and 25 °C. However there was observed an increasing trend when stored at 4 °C and decreasing when at 25 °C. It can be concluded that stored at lower temperature ensures better protection of active ingredients present in obtained powders by the carrier material. At higher temperature (25 °C) some degradation of carrier material at the surface could take place, and some of the active ingredients (anthocyanins, polyphenols) could be exposed to oxidation. The same observation for anthocyjanin level in storage powders was made by Moser et al. (2017) for anthocyanins from mango juice powders but it was different for stored chokeberry powders with MA:AG obtained by Pieczykolan and Kurek (2019), in which no changes were observed.

### Polyphenols measurement

The juice contained over 8888 + 274 mg/100 g d.m. polyphenols. Spray drying caused a massive decrease of this value, to about 3000 mg/100 g d.m. Higher content was obtained by Horszwald et al. (2013); polyphenols content in chokeberry powder were 34,280 mg/100 g. Similar values for chokeberry powders based on MD with different DE were presented by Gawałek et al. (2017). The lowest amount of polyphenols was recorded for powders containing only maltodextrin, independently of dextrose equivalent. There was no statistically significant impact of change of DE and drying temperature on the polyphenol content (Table 3). Bakowska-Barczak and Kolodziejczyk (2011) drying blackcurrant obtained a similar relationship when testing the impact of carrier on polyphenol content, and the opposite relationship when measuring the impact of temperature on polyphenols content. The opposite tendency for the temperature-polyphenol relationwas presented by Gawałek et al. (2017), while a similar tendency was observed for increasing DE in maltodextrin.

A statistically significant impact of storage temperature on polyphenols content was observed only for powders based on MD10, MD15 (160 °C), AG:MD15 1:1 (160 °C) and AG:MD15 3:1 (200 °C) (Table 3). During storage the content of polyphenols in those samples increased, regardless of storage temperature. A similar relationship was recorded when storing blackcurrant powders at 8 °C for 3 months (Bakowska-Barczak and Kolodziejczyk 2011). However that increase could be related to the method used, in which also sugar could be detected (Śledź and Witrowa-Rajchert 2012).

In almost all powders it was observed, that the the polyphenol content was lower in powders stored at 25 °C than in powders stored at 4 °C, but those differences were statistically insignificant. Polyphenol content in samples varied significantly, but no trend was observed. The highest contents of polyphenols during storage at 4 °C and 25 °C were recorded for AG 200 °C and AG:MD15 3:1 200 °C, respectively, and the lowest for MD10 200 °C and MD10 160 °C, respectively (Table 3). In most cases there was no statistically significant impact of drying temperature on polyphenols content. The higher polyphenols content during storage might be related to creation of compounds that are not polyphenols, but react with the reagent used for detection of polyphenol compounds. The result might also be overstated due to components, such as sugars and ascorbic acid, because the method used for polyphenols detection is not very specific (Śledź and Witrowa-Rajchert 2012).

### Correlations

**Fig. 3:**
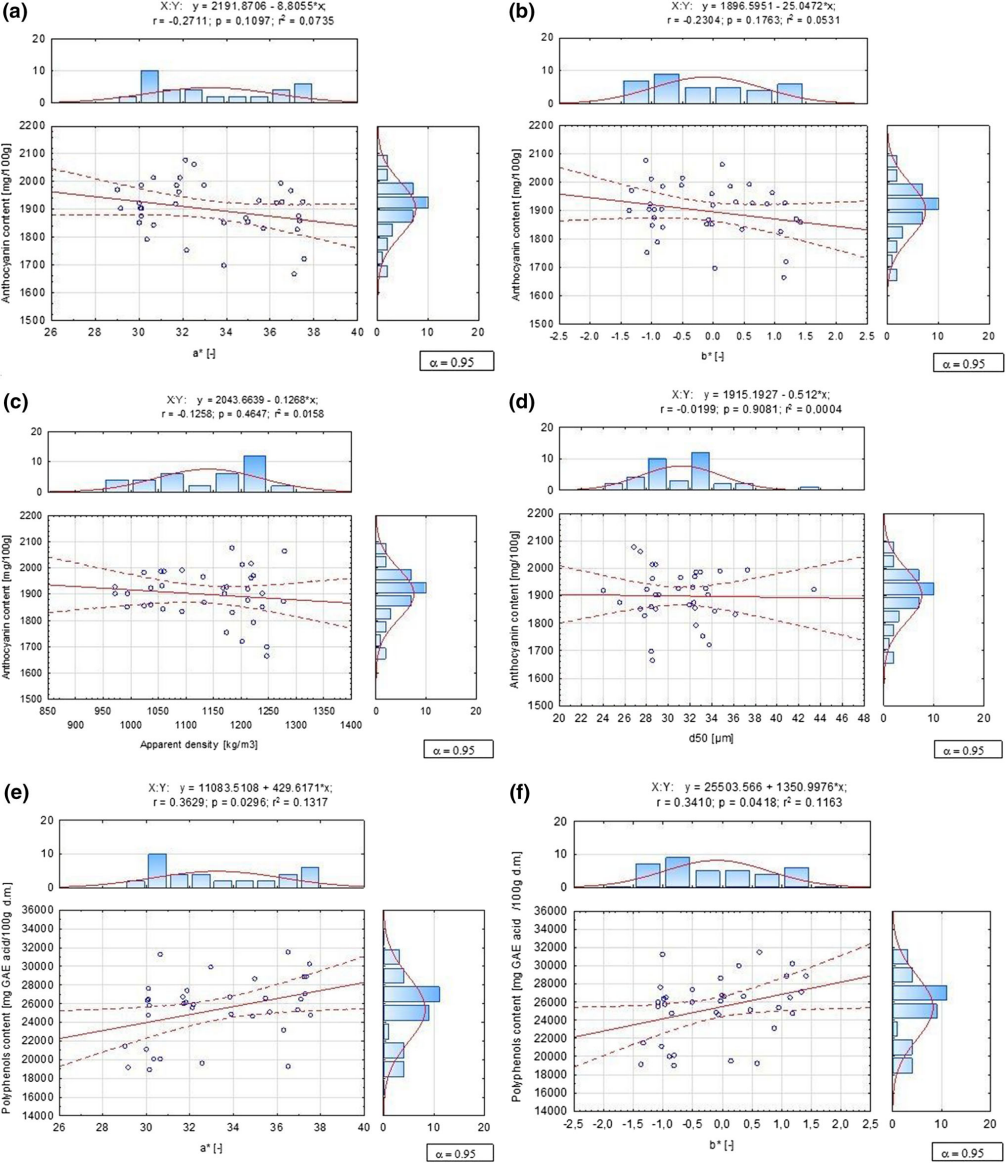
Correlation between **a** anthocyjanin content—a*, **b** anthocyjanin content—b*, **c** anthocyjanin content—apparent density, **d** anthocyjanin content-d_50_, **e** polyphenols content—a*, **f** polyphenols content—b*

Linear Pearson correlation was determined between the content of anthocyanins and polyphenols, and the colour parameters a* and b*, and between anthocyanins content, and apparent density and particles diameter (Fig. 3). No statistically significant correlation for anthocyjanin was seen, for all correlation r was below significant value from Pearson table (n = 36–2, r = 0.3246). However for polyphenols correlation with colour coefficient a* (y = 4924.01 − 62.45*x; r = -0.4161; *p* = 0.0116; r^2^ = 0.1731) and b* (y = 3007.33–366.64*x; r = -0.7647; *p* = 0.000; r^2^ = 0.5848) was seen. With good precision it could be calculated from presented equations.

## Conclusion

Used drying parameters and carrier materials allowed us to create powders with satisfactory quality and stability, because they were characterized by low water activity (< 0.26), high dry matter content (97–99%) and good hygroscopic properties. It was demonstrated that dryer inlet temperature and used carrier type had a statistically significant impact on some of the physicochemical properties of powders. Higher drying temperature caused creation of a smoother surface of particles, lower particles diameter, lower apparent density, smaller water activity and higher polyphenols content, but it also decreased anthocyanins content and increased diffusion properties. Powders containing high concentration of arabic gum were characterized by lower water activity and lower apparent density, although they caused decrease of colour parameters values and increase of diffusion properties (at a_w_ < 0.5). The mixtures of carriers, containing arabic gum and maltodextrin, appeared to have a large potential to ensure a higher quality of chokeberry powders. It was demonstrated that the storage temperature of powders had no statistically signifficant impact on examined physicochemical properties, but a trend was observed. The contents of anthocyanins and polyphenols were higher in powders stored at 4 °C.

## Electronic supplementary material

Below is the link to the electronic supplementary material.

Supplementary material 1 (DOCX 31 kb)
