# Supplementary material for: The influence of spray drying parameters and carrier material on the physico-chemical properties and quality of chokeberry juice powder
Source: J Food Sci Technol. 2019 Sep 12;57(2):564–77. doi: 10.1007/s13197-019-04088-8 (PMC7016068; doi:10.1007/s13197-019-04088-8)
Supplement: Supplementary file 1 — Supplementary material 1 (DOCX 31 kb) [file 13197_2019_4088_MOESM1_ESM.docx]

Table. S1. Hygroscopicity for chokeberry powders and coefficients for equation 1

| Carrier type | Hygroscopicity after 144h  (g H20/ g d.m.) | Coefficients of equation | | | r^2^ | RMSE | | Equilibrium value |
| --- | --- | --- | --- | --- | --- | --- | --- | --- |
|  |  | a | b | c |  | (%) | |  |
|  |  | 0.225 | | |  |  | |  |
| MD10 160^o^C | 0.0236 | 0.0026 | 0.028 | 0.698 | 0.971 | 0.114 | | 0.031 |
| MD10 200^o^C | 0.0272 | 0.0031 | 0.030 | 0.881 | 0.971 | 0.134 | | 0.033 |
| MD15 160^o^C | 0.0313 | 0.0033 | 0.034 | 1.029 | 0.978 | 0.145 | | 0.037 |
| MD15 200^o^C | 0.0255 | 0.0009 | 0.030 | 0.981 | 0.987 | 0.086 | | 0.031 |
| GA:MD10 1:3 160^o^C | 0.0315 | 0.0020 | 0.038 | 0.642 | 0.987 | 0.102 | | 0.040 |
| GA:MD10 1:3 200^o^C | 0.0337 | 0.0020 | 0.396 | 0.697 | 0.988 | 0.115 | | 0.398 |
| GA:MD10 1:1 160^o^C | 0.0312 | 0.0017 | 0.041 | 0.417 | 0.990 | 0.105 | | 0.043 |
| GA:MD10 1:1 200^o^C | 0.0398 | 0.0028 | 0.048 | 0.506 | 0.987 | 0.133 | | 0.051 |
| GA:MD10 3:1 160^o^C | 0.0243 | 0.0028 | 0.037 | 0.264 | 0.966 | 0.114 | | 0.040 |
| GA:MD10 3:1 200^o^C | 0.0321 | 0.0047 | 0.035 | 0.682 | 0.932 | 0.229 | | 0.040 |
| GA:MD15 1:3 160^o^C | 0.0244 | 0.0027 | 0.032 | 0.469 | 0.965 | 0.121 | | 0.035 |
| GA:MD15 1:3 200^o^C | 0.0302 | 0.0033 | 0.033 | 0.975 | 0.960 | 0.172 | | 0.036 |
| GA:MD15 1:1 160^o^C | 0.0322 | 0.0033 | 0.041 | 0.393 | 0.973 | 0.141 | | 0.044 |
| GA:MD15 1:1 200^o^C | 0.0362 | 0.0038 | 0.042 | 0.588 | 0.971 | 0.171 | | 0.046 |
| GA:MD15 3:1 160^o^C | 0.0306 | 0.0031 | 0.042 | 0.319 | 0.972 | 0.135 | | 0.045 |
| GA:MD15 3:1 200^o^C | 0.0348 | 0.0034 | 0.042 | 0.524 | 0.970 | 0.166 | | 0.045 |
| GA 160^o^C | 0.0330 | 0.0033 | 0.041 | 0.429 | 0.971 | 0.154 | | 0.044 |
| GA 200^o^C | 0.0356 | 0.0038 | 0.042 | 0.501 | 0.965 | 0.189 | | 0.046 |
|  |  | 0.4 | | |  |  | |  |
| MD10 160^o^C | 0.4405 | 0.0022 | 0.052 | 0.570 | 0.987 | 0.191 | | 0.054 |
| MD10 200^o^C | 0.0503 | 0.0017 | 0.057 | 0.679 | 0.995 | 0.136 | | 0.059 |
| MD15 160^o^C | 0.0538 | 0.0017 | 0.062 | 0.609 | 0.995 | 0.143 | | 0.064 |
| MD15 200^o^C | 0.0517 | 0.0013 | 0.060 | 0.630 | 0.995 | 0.129 | | 0.061 |
| GA:MD10 1:3 160^o^C | 0.0573 | 0.0012 | 0.068 | 0.482 | 0.995 | 0.185 | | 0.069 |
| GA:MD10 1:3 200^o^C | 0.0587 | 0.0011 | 0.069 | 0.517 | 0.997 | 0.134 | | 0.070 |
| GA:MD10 1:1 160^o^C | 0.0593 | 0.0007 | 0.074 | 0.361 | 0.992 | 0.230 | | 0.075 |
| GA:MD10 1:1 200^o^C | 0.0657 | 0.0005 | 0.080 | 0.402 | 0.994 | 0.224 | | 0.081 |
| GA:MD10 3:1 160^o^C | 0.0604 | 0.0021 | 0.076 | 0.313 | 0.978 | 0.366 | | 0.078 |
| GA:MD10 3:1 200^o^C | 0.0578 | 0.0018 | 0.072 | 0.341 | 0.960 | 0.743 | | 0.074 |
| GA:MD15 1:3 160^o^C | 0.0563 | 0.0019 | 0.071 | 0.322 | 0.949 | 0.840 | | 0.073 |
| GA:MD15 1:3 200^o^C | 0.0557 | 0.0019 | 0.070 | 0.328 | 0.952 | 0.263 | | 0.072 |
| GA:MD15 1:1 160^o^C | 0.0579 | 0.0019 | 0.073 | 0.312 | 0.961 | 0.308 | | 0.075 |
| GA:MD15 1:1 200^o^C | 0.0585 | 0.0021 | 0.074 | 0.294 | 0.965 | 0.169 | | 0.076 |
| GA:MD15 3:1 160^o^C | 0.0559 | 0.0020 | 0.071 | 0.302 | 0.952 | 0.422 | | 0.073 |
| GA:MD15 3:1 200^o^C | 0.0564 | 0.0021 | 0.073 | 0.280 | 0.955 | 0.245 | | 0.075 |
| GA 160^o^C | 0.0567 | 0.0022 | 0.074 | 0.256 | 0.955 | 0.393 | | 0.076 |
| GA 200^o^C | 0.0570 | 0.0024 | 0.076 | 0.240 | 0.957 | 0.314 | | 0.078 |
|  |  | 0.6 | | |  |  | |  |
| MD10 160^o^C | 0.1014 | 0.0018 | 0.119 | 0.307 | 0.998 | 0.517 | | 0.121 |
| MD10 200^o^C | 0.1014 | 0.0018 | 0.119 | 0.307 | 0.998 | 0.258 | | 0.121 |
| MD15 160^o^C | 0.0947 | -0.0002 | 0.109 | 0.414 | 1.000 | 0.067 | | 0.109 |
| MD15 200^o^C | 0.1088 | 0.0019 | 0.138 | 0.172 | 0.993 | 0.305 | | 0.140 |
| GA:MD10 1:3 160^o^C | 0.1147 | 5.7e-05 | 0.147 | 0.169 | 1.000 | 0.040 | | 0.147 |
| GA:MD10 1:3 200^o^C | 0.1129 | -0.0017 | 0.147 | 0.165 | 0.999 | 0.133 | | 0.145 |
| GA:MD10 1:1 160^o^C | 0.1296 | 0.0004 | 0.177 | 0.105 | 1.000 | 0.267 | | 0.177 |
| GA:MD10 1:1 200^o^C | 0.1318 | -5.6e-05 | 0.166 | 0.162 | 1.000 | 0.097 | | 0.166 |
| GA:MD10 3:1 160^o^C | 0.0846 | 0.0019 | 0.108 | 0.209 | 0.996 | 0.204 | | 0.110 |
| GA:MD10 3:1 200^o^C | 0.1004 | 0.0026 | 0.121 | 0.238 | 0.997 | 0.212 | | 0.124 |
| GA:MD15 1:3 160^o^C | 0.0748 | 0.0021 | 0.093 | 0.274 | 0.997 | 0.140 | | 0.095 |
| GA:MD15 1:3 200^o^C | 0.0830 | 0.0022 | 0.099 | 0.319 | 0.997 | 0.163 | | 0.101 |
| GA:MD15 1:1 160^o^C | 0.0917 | 0.0020 | 0.112 | 0.248 | 0.998 | 0.155 | | 0.114 |
| GA:MD15 1:1 200^o^C | 0.0955 | 0.0025 | 0.114 | 0.269 | 0.998 | 0.156 | | 0.117 |
| GA:MD15 3:1 160^o^C | 0.0897 | 0.0011 | 0.116 | 0.191 | 0.998 | 0.152 | | 0.117 |
| GA:MD15 3:1 200^o^C | 0.0922 | 0.0013 | 0.116 | 0.218 | 0.998 | 0.161 | | 0.117 |
| GA 160^o^C | 0.0977 | 0.0018 | 0.119 | 0.248 | 0.997 | 0.190 | | 0.121 |
| GA 200^o^C | 0.1014 | 0.0018 | 0.119 | 0.307 | 0.998 | 0.181 | | 0.121 |
|  |  |  | 0.75 |  |  |  | |  |
| MD10 160^o^C | 0.1137 | 0.0122 | 0.132 | 0.176 | 0.968 | 0.549 | 0.144 | |
| MD10 200^o^C | 0.1092 | 0.0036 | 0.139 | 0.161 | 0.996 | 0.173 | 0.143 | |
| MD15 160^o^C | 0.0941 | 0.0002 | 0.114 | 0.288 | 0.999 | 0.189 | 0.114 | |
| MD15 200^o^C | 0.0880 | 0.0014 | 0.100 | 0.467 | 0.997 | 0.138 | 0.101 | |
| GA:MD10 1:3 160^o^C | 0.1262 | 0.0019 | 0.176 | 0.095 | 0.999 | 0.131 | 0.178 | |
| GA:MD10 1:3 200^o^C | 0.1245 | 0.0018 | 0.166 | 0.118 | 0.999 | 0.235 | 0.168 | |
| GA:MD10 1:1 160^o^C | 0.1546 | 0.0020 | 0.276 | 0.031 | 0.999 | 0.164 | 0.278 | |
| GA:MD10 1:1 200^o^C | 0.1562 | 0.0027 | 0.242 | 0.050 | 0.998 | 0.154 | 0.245 | |
| GA:MD10 3:1 160^o^C | 0.0865 | 0.0009 | 0.118 | 0.156 | 0.998 | 0.168 | 0.119 | |
| GA:MD10 3:1 200^o^C | 0.0991 | 0.0018 | 0.127 | 0.179 | 0.998 | 0.174 | 0.129 | |
| GA:MD15 1:3 160^o^C | 0.0793 | 0.0010 | 0.101 | 0.236 | 0.999 | 0.091 | 0.102 | |
| GA:MD15 1:3 200^o^C | 0.0871 | 0.0017 | 0.103 | 0.335 | 0.998 | 0.155 | 0.105 | |
| GA:MD15 1:1 160^o^C | 0.0926 | 0.0014 | 0.123 | 0.164 | 0.997 | 0.205 | 0.124 | |
| GA:MD15 1:1 200^o^C | 0.0979 | 0.0016 | 0.122 | 0.210 | 0.998 | 0.153 | 0.124 | |
| GA:MD15 3:1 160^o^C | 0.0916 | 0.0011 | 0.119 | 0.185 | 0.998 | 0.132 | 0.120 | |
| GA:MD15 3:1 200^o^C | 0.1016 | 0.0013 | 0.124 | 0.243 | 0.999 | 0.140 | 0.125 | |
| GA 160^o^C | 0.0997 | 0.0009 | 0.135 | 0.142 | 0.997 | 0.189 | 0.136 | |
| GA 200^o^C | 0.1020 | 0.0015 | 0.126 | 0.219 | 0.999 | 0.123 | 0.128 | |

a,b,c,d -values with different letters in the same column differ significantly (p < 0.05).
